# Automated image analysis of nuclear shape: What can we learn from a prematurely aged cell?

**DOI:** 10.18632/aging.100434

**Published:** 2012-02-16

**Authors:** Meghan K. Driscoll, Jason L. Albanese, Zheng-Mei Xiong, Mitch Mailman, Wolfgang Losert, Kan Cao

**Affiliations:** ^1^ Department of Physics, University of Maryland, College Park, MD 20742, USA; ^2^ Department of Cell Biology and Molecular Genetics, University of Maryland, College Park, MD 20742, USA

**Keywords:** progeria, aging, rapamycin, mTOR, nucleus

## Abstract

The premature aging disorder, Hutchinson-Gilford progeria syndrome (HGPS), is caused by mutant lamin A, which affects the nuclear scaffolding. The phenotypic hallmark of HGPS is nuclear blebbing. Interestingly, similar nuclear blebbing has also been observed in aged cells from healthy individuals. Recent work has shown that treatment with rapamycin, an inhibitor of the mTOR pathway, reduced nuclear blebbing in HGPS fibroblasts. However, the extent of blebbing varies considerably within each cell population, which makes manual blind counting challenging and subjective. Here, we show a novel, automated and high throughput nuclear shape analysis that quantitatively measures curvature, area, perimeter, eccentricity and additional metrics of nuclear morphology for large populations of cells. We examined HGPS fibroblast cells treated with rapamycin and RAD001 (an analog to rapamycin). Our analysis shows that treatment with RAD001 and rapamycin reduces nuclear blebbing, consistent with blind counting controls. In addition, we find that rapamycin treatment reduces the area of the nucleus, but leaves the eccentricity unchanged. Our nuclear shape analysis provides an unbiased, multidimensional “fingerprint” for a population of cells, which can be used to quantify treatment efficacy and analyze cellular aging.

## INTRODUCTION

Hutchinson-Gilford progeria syndrome (HGPS) is a rare genetic disease that occurs in approximately 1 out of 4 million live births [1]. Visible symptoms of patients with HGPS include a pronounced forehead, short stature, receding mandible, conspicuous veins in the scalp, alopecia and diminished subcutaneous fat [1-4]. Internally, such patients undergo accelerated organ degeneration. The average life expectancy of HGPS patients is just 14 years, with death typically resulting from heart attacks or stroke [1-4].

The genetic mutation that leads to HGPS occurs in exon 11 of the human *LMNA* gene, which plays a role in nuclear scaffolding [5, 6]. This HGPS mutation is a *de novo* single nucleotide substitution (1824 C => T), which does not change the amino acid coding sequence [GGC (glycine) => GGT (glycine)]. However, this mutation partially activates a cryptic splice donor site, which causes a 150-nucleotide sequence to be spliced out of exon 11 and leads to the production of the mutant protein progerin, also known as LAΔ50 [7]. Because of this internal deletion, progerin does not contain the cleavage site required for the removal of the farnesyl group by protease Zempste 24, so the farnesyl group remains attached to progerin [1, 8]. The farnesyl chain is hydrophobic and has a strong affinity for the inner nuclear membrane. As a result, progerin abnormally inserts into the nuclear membrane, resulting in bulging of the nuclear envelope. This abnormal nuclear shape, commonly referred to as “nuclear blebbing”, has been the hallmark cellular phenotype for HGPS cells [1, 8], yet the molecular and physical mechanisms of nuclear blebbing are not well understood. In addition, the presence of progerin results in alterations in histone methylation, a thickened nuclear lamina, genome instability, clustering of nuclear pores, and loss of heterochromatin [9]. As progerin continues to build up inside prematurely aged cells, the nuclear blebbing phenotype and other damaging effects become more severe [9]. Cellular division is also affected in HGPS cells: during mitosis, when the nuclear envelope disassembles, the progerin forms aggregates with membranes, interferes with nuclear membrane disassembly, and mislocalizes to the cytoplasm after mitosis, leading to chromosome mis-segregation and binucleation [10, 11].

Much work has also been done in an effort to develop a cure for HGPS. Children with HGPS are currently participating in the first clinical trial, testing a drug therapy that uses farnesyl transferase inhibitors (FTIs), which block the addition of the farnesyl group to progerin (Progeria Research Foundation 2011)[8, 12-14]. More recently, we showed that the macrolide antibiotic rapamycin can reverse the nuclear blebbing and other phenotypes in HGPS cells through down-regulating progerin, which suggests its potential as a treatment for HGPS [15-17]. In both FTI and rapamycin studies, the percentages of nuclear blebbing, as scored by blind observers, were used as the first indication of the effectiveness of the drugs. However, it is not possible to define whether a cell is blebbed unambiguously because many cells in both healthy and diseased populations contain minor abnormalities in nuclear shape. Hence, the fraction of cells counted as blebbed can vary considerably among different observers, making blebbing quantification an inherently statistical problem.

A number of studies have suggested a strong connection between HGPS and the normal aging processes. In 2006, Misteli's group reported the detection of progerin mRNA and protein in cells obtained from healthy individuals, indicating that the cryptic splice site in exon 11 is also used in the presence of the normal sequence of exon 11 [18]. Similar to the results describe above, we detected low levels of progerin in normal cells, and a significant percentage of these cells had mitotic defects similar to those found in HGPS cells [10]. Our recent study further revealed a causative connection between dysfunctional telomeres and the cryptic splicing of lamin A [19]. Moreover, studies using tissues taken from normal human subjects revealed that at any age, the cryptic splicing event takes place in skin, and as we grow older, progerin-positive fibroblasts become more abundant [20, 21]. Thus, one may expect a broad distribution in the severity of blebbing in a normal cell population and an increase in blebbing with aging.

Here, we report an automated, quantitative method that we used suitable to study distributions of blebbing in a large cell population. In this method, the nuclear morphology, as visualized by immunofluorescence staining of lamin A/C, is quantified using image analysis software that extracts the nuclear boundary from microscopy images and then calculates measures such as area, perimeter, and curvature for each nucleus. For each set of treated cells, the curvature of all of the nuclei can further be visualized in a single plot. Since curvature is a mathematically complete description of shape, these plots allow for the quick assessment of the severity of blebbing in a population of nuclei. We applied our method to examine HGPS fibroblasts treated with either mock, rapamycin, or RAD001, a derivative of rapamycin with better tolerance in patients. We found that treatment with RAD001 or rapamycin decreases both blebbing and nuclear area in a dosage dependent manner, but leaves nuclear eccentricity unchanged. Our study presents a novel, unbiased, quantitative method for analyzing HGPS and aging cells. This method could be useful for future drug screenings for HGPS or other age related diseases, patient diagnostics, and quantitative modeling of nuclear shape.

## RESULTS

### The nuclear shape of HGPS and normal fibroblast cells can be automatically extracted, visualized, and analyzed

**Figure 1:**
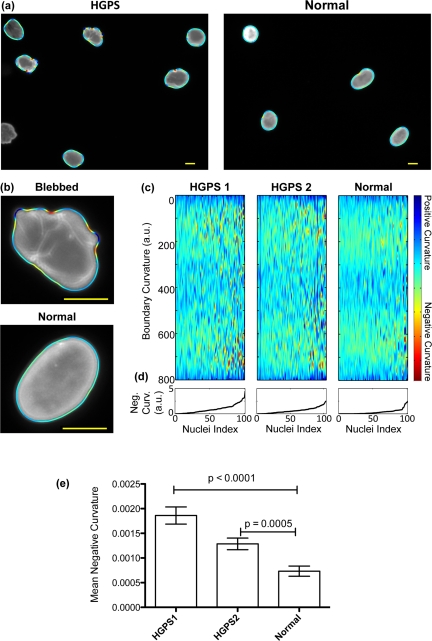
The boundary curvature of HGPS and normal nuclei (**a**) The curvature of nuclei is automatically extracted from fluorescence images of anti-laminA/C immunostaining. Here, the curvature of HGPS and normal nuclei is shown as a colored outline, where blue represents regions of large positive curvature, and red regions of large negative curvature (scale bar: 10 μm). Blebbed nuclei have more regions of negative curvature, and so have more red signals. (**b**) High magnification examples of the extracted boundary curvature of a blebbed, HGPS nucleus, and a more oval, normal nucleus (scale bar: 10 μm). (**c**) The boundary curvature of hundreds of nuclei can be represented in a single heat map. In these heat maps, which here correspond to two HGPS cell lines (HGPS1 and HGPS2, respectively) and one control cell line (Normal), each vertical line is the stretched, colored outline of a single nucleus. Regions of large negative curvature are colored blue while regions of large negative curvature are colored red. (**d**) The nuclei are ordered from left to right by increasing mean negative curvature (MNC), which is shown in the line plots. (**e**) The MNC of populations from both HGPS cell lines is statistically different from the population from the normal control, as illustrated in this histogram.

In order to test our automatic analysis of nuclear shape, we first cultured fibroblasts from two HGPS fibroblast cell lines (HGADFN155-p15 and HGADFN167-p12, HGPS1 and HGPS2 respectively) and from one normal control (HGFDFN168-p14, normal). The cells were fed with fresh MEM medium containing 15% FBS and grown at 37°C. To visualize the nuclei, we performed immunofluorescence staining of the nuclear membrane with a mouse monoclonal antibody raised against lamin A/C (MAB3211). This antibody has been well characterized in HGPS cells and has also been used in studies on other laminopathies. Fluorescence images of about 100 randomly selected nuclei per cell line were taken with a Zeiss fluorescence microscope at 400X magnification (examples are shown in Figure 1a). A custom-written MATLAB program (details as described in Material and Methods) was used to extract nuclear shapes and properties of the shape, such as boundary curvature. In Figures 1a and b, the nuclear boundaries, which are colored by curvature, are shown overlaid on the microscopy images. Convex curvatures were kept positive, while concave curvatures were made negative. As shown by the color bar in Figure 1c, blue represents regions of large positive curvature, and red represents regions of large negative curvature. A blebbed cell, such as the cell shown in the top of Figure 1b, will have boundary regions that are dark red and dark blue, whereas a non-blebbed cell, such as the cell shown in the bottom of Figure 1b, will have boundary regions that are mostly blue and green with almost no red.

We next assembled plots that, for each cell line, display the boundary curvatures of all of the measured nuclei, as shown in Figure 1c. In these heat maps, each vertical line represents the boundary curvature of one nucleus. To construct such a plot, imagine cutting each colored boundary at the location farthest from the nucleus center (that location is labeled 0 on the heat map), pulling the boundary straight, and then lining it up next to the colored boundaries of the other nuclei. The heat maps of blebbed populations, such as the HGPS cell lines, have many red speckles, whereas the heat maps of unblebbed populations, such as the control cell line, have few red speckles.

Within each plot, the nuclei are ordered from left to right by increasing mean negative curvature (MNC), a measure of nuclear blebbing. We defined the MNC of each nucleus by averaging all negative curvatures, excluding the positive curvatures entirely, and taking the absolute value. As shown in Figures 1d and 1e, the HGPS1 and HGPS2 cell lines have larger MNCs, and hence are more blebbed, than the control cell line. HGPS1 also has a larger MNC than HGPS2, perhaps because HGPS1 is at a later cellular passage, and thus more senesced. We found that both HGPS MNC distributions are statistically different from the MNC distribution of the control (see Figure 1e for p-values).

To validate the automated nuclear shape analysis, we also assessed nuclear morphology using the standard technique: manual blind counting. Nuclei with protrusions or invaginations were counted as blebed, while other nuclei were counted as normal. We found that 73% of HGPS1 nuclei, 63% of HGPS2 nuclei, and 24% of normal nuclei were abnormal. These counts are in quantitative agreement with the MNC distributions of the respective cell lines (Figure 1e). In order to better evaluate how the results of manual counting correlate with quantitative shape metrics, we had experienced human counters label individual nuclei as either normal or blebbed, and analyzed the MNC of the two populations. The analysis shows a positive correlation between MNC and the percentage of nuclei that were labeled as blebbed (Figure S1).

Since the automated analysis extracts the boundary of each nucleus, we can assess nuclear morphology using various shape metrics besides boundary curvature. For each nucleus, we also calculated area, perimeter, number of invaginations, eccentricity and other metrics. In analogy to how microarray data is analyzed to find relationships between genes, we used correlation as a measure of interrelationship between the 15 different measures of nuclear shape determined in this study. We hierarchically clustered the 15 measures of nuclear shape and laminA/C fluorescence intensity (Figure S2). We found several families of nuclear measures that roughly correspond to size, extent of blebbing, eccentricity, laminA/C fluorescence intensity, and the standard deviation of fluorescence intensity. The clustering reassures us that the intensity, which is affected by immunostaining and imaging details, does not notably affect the measured MNC. The clustering also indicates that the standard deviation in MNC and the tortuosity are measures related to MNC. Also related to mean MNC is the solidity, which is the ratio of the measured area and the area of ‘convex hull,’ or the minimal convex shape that bounds the measured shape of the nucleus.

As a control experiment, we tested whether the cell density would influence the MNC. We seeded cells from the same HGPS cell line at densities of 3000, 9000, and 27000 cells per well in 4-well chamber slides (Figure S3). The three densities did not appear to have different MNC distributions, nor were the measured MNC distributions statistically distinct.

### RAD001 and rapamycin similarly reduce blebbing in HGPS fibroblast cells

Recent work has revealed that rapamycin, an mTOR (mammalian target of rapamycin) inhibitor, significantly reduces the phenotypic hallmarks of progeria in HGPS cells by down-regulating progerin [15]. Everolimus (RAD001), which is the 40-O-(2-hydroxyethyl) derivative of rapamycin, works similarly to sirolimus as an mTOR inhibitor but is better tolerated by patients. In order to compare the efficacy of RAD001 to rapamycin, we treated HGPS fibroblasts with rapamycin, RAD001, or mock, and then analyzed the nuclear morphology of each treatment group.

**Figure 2:**
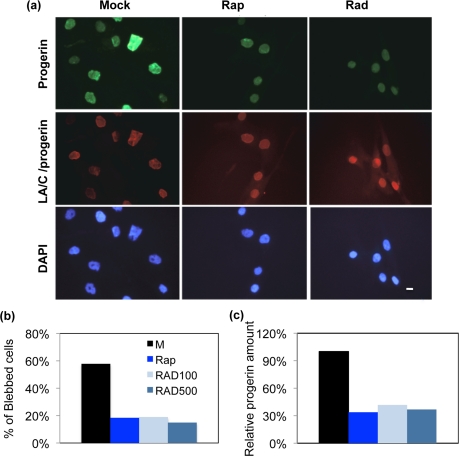
The nuclear morphology and progerin levels of rapamycin and RAD001 treated HGPS cells (**a**) The phenotype of nuclear blebbing was improved in RAD001 and rapamycin treated HGPS fibroblast cells. Cells were stained with DAPI (blue), laminA/C antibody (red), and progerin antibody (green) to show nuclear location and morphology. The treatment duration is for seven weeks. Mock: vehicle (DMSO, 0.025% v/v); Rap: 0.68 μM rapamycin, Rad: 0.1 μM RAD001. (Scale bar: 10 μm) (**b**) Quantification of the percentage of blebbing in all treatments. At least 200 nuclei were counted blindly. M: vehicle (DMSO, 0.025% v/v); Rap: 0.68 μM rapamycin; RAD100: 0.1 μM RAD001 treatment; RAD500: 0.5 μM RAD001 treatment. (**c**) Progerin was decreased in rapamycin or RAD001 treated HGPS fibroblasts. The relative amount of progerin was quantified using quantitative western blotting analysis and compared to the mock-treated HGPS cells.

Cultured fibroblasts from an HGPS patient (HGADFN155-p15) and a normal individual (HGFDFN168-p15) were used in this experiment. The cells were fed every other day with fresh MEM medium containing 0.68 μM rapamycin, 0.1 μM RAD001, 0.5 μM RAD001, or the same volume of vehicle (DMSO, 0.025% v/v) for a duration of seven weeks. To examine the effects on nuclear morphology, we labeled cells with an antibody for lamin A/C and an antibody specific for progerin (Figure 2a). To judge the impact of rapamycin and RAD001, we first scored the percentage of nuclei with abnormal morphology in the usual way by manual blind counting. At least 200 randomly selected cells were scored by fluorescence microscopy for each cell line under each condition. In comparison with the passage-matched, mock-treated HGPS cells, the rapamycin or RAD001 treated HGPS cells exhibited a clear reduction in nuclear blebbing (Figure 2b). Since increased genome instability was reported in HGPS cells [15, 22, 23], we also examined whether RAD001 treatment can improve this phenotype. Using immunofluorescence staining, we observed a reduction in 53BP1 foci in rapamycin or RAD001 treated cells, indicating that inhibition of mTOR prevents DNA damage induced in prematurely senescent cells by progerin (Figure S4). Quantification of progerin protein by western blotting analysis also revealed an over 50% reduction in progerin levels in rapamycin and RAD001 treated HGPS cells (Figure 2c). We also detected a weaker progerin-staining signal in almost all of the rapamycin or RAD001 treated HGPS cells, and their nuclear morphology appeared substantially improved compared to untreated cells (Figure 2a).

As reported in Cao et al. [15], we again noticed a reduction in overall cell proliferation at the beginning of the treatments (less than two weeks) with either rapamycin or RAD001 compared to the mock treated samples. We used a tetrazolium salt-based cell proliferation assay (WST1 assay) to analyze this apparent cell growth inhibition for the concentrations of RAD001 used in the experiment: 0, 20, 60, 100, and 500 nM. Figure S5 shows that all treatments for both control and HGPS cell lines had a similar reduction in cell proliferation compared to the mock treatments (less than 40%), suggesting that any effective dose of RAD001 may have similar anti-hypertrophic effects [24].

**Figure 3:**
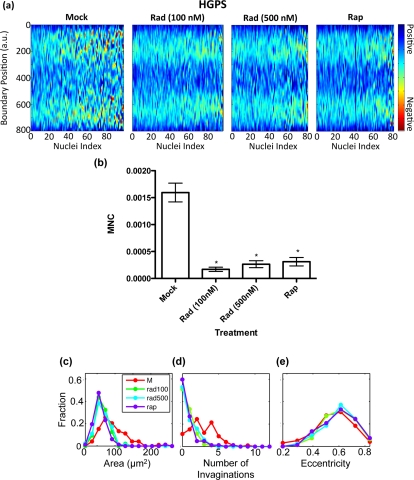
Imaging analysis of RAD001 and rapamycin treated HGPS cells (**a**) Heat maps that represent the boundary curvature of the HGPS cells treated with the vehicle (mock), 100 nM and 500 nM RAD001 (Rad), and rapamycin (Rap). (**b**) MNC of populations from the mock treated HGPS cell lines is statistically different from the populations of RAD001 or rapamycin treated cells (*p* < 0.001) (**c-e**) Compared to the mock treated nuclei, the RAD001 and rapamycin treated nuclei have smaller area (**c**) and fewer invaginations (**d**), but similar eccentricity (**e**).

In parallel to the blind counting, we took immunofluorescence images of about 100 randomly selected nuclei per treatment group and automatically analyzed their nuclear morphology. Heat maps, which display the boundary curvature of the treated HGPS cells, are shown in Figure 3a. From the heat maps we see that the mock treated cells are much more blebbed than the rapamycin or RAD001 treated cells, which is consistent with our blinded counting. Indeed, we found that the MNC distributions of the rapamycin and RAD001 treated cells were statistically different from that of the control group (Figure 3b). Similarly, our analysis showed a reduction in the number of invaginations in treated HGPS cells (Figure 3d).

Interestingly, we also found that the RAD001 and rapamycin treated nuclei had a smaller area than the mock treated nuclei (Figure 3c). Moreover, we noticed that the eccentricity, which is a measure of how elongated the nuclei are, did not change as a result of the rapamycin or RAD001 treatments (Figure 3e). Our analysis suggested that rapamycin or RAD001 treatments appear to locally improve abnormal morphology, without affecting the overall shape of the nuclei, though still altering nuclear size (see discussion). In summary, our data suggest that, similar to rapamycin, RAD001 can reverse the nuclear phenotypes in HGPS cells through promoting progerin clearance.

### RAD001 induces a gradual change in nuclear morphology in a dosage dependant manner

**Figure 4:**
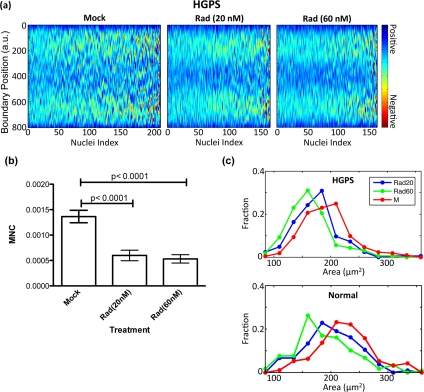
RAD001 induces a gradual change in nuclear morphology in a dosage dependant manner (**a**) Heat maps that represent the boundary curvature of HGPS cells treated with the vehicle (mock) or 20 or 60 nM RAD001. (**b**) Both doses significantly reduce the mean negative curvature as shown by the histogram. (**c**) The area of mock treated nuclei is greater than both doses of RAD001 treated nuclei, but the nuclei that received the smaller dose of RAD001 have greater area than the nuclei that received the larger dose. The same trend in area change is apparent to the same extent in the treated control normal fibroblasts.

Based on the above analysis, we suggested RAD001 could be used at 100 nM concentration to achieve similar beneficial effects in HGPS cell cultures as rapamycin at 0.68 μM as described in Cao et al. [15]. Next, we explored the sensitivity of the curvature analysis program, since quantitative image analysis is most useful if it can reveal small changes that are difficult to observe. Thus, we lowered the dosage of RAD001 to 20 or 60 nM, and shortened the duration of treatment to two weeks. An HGPS fibroblast cell line (HGADFN167-p15) and a control fibroblast cell line (HGFDFN168-p15) were fed with fresh MEM medium containing 20 nM RAD001, 60nM RAD001 or the same volume of vehicle (0.3% DMSO) every other day. Nuclear curvature outline and heat map analyses of MNC were carried out at the end of the two-week treatment (Figure 4a). Box plot analysis indicated a significant reduction of MNC in the HGPS cell line, even in the cells receiving 20 nM RAD001 (Figure 4b), while those minor morphological improvements were not visible with the traditional blinded counting method, suggesting that the automated analysis is more sensitive. Importantly, we noticed a dosage dependent reduction in area of the nuclei of both treated HGPS and treated control cells (Figure 4c): the area of mock treated nuclei was greater than both doses of RAD001 treated nuclei, but the nuclei that received the smaller dose of RAD001 (20 nM) had greater area than the nuclei that received the larger dose (60 nM). This result suggests the improvement in nuclear shape is a gradual process, the area reduction is primarily due to non-specific effects of the drug treatment, and incrementtal improvement during treatment can be captured and quantified by this curvature outline imaging analysis.

## DISCUSSION

One of the hallmarks of HGPS is the abnormal nuclear shape known as blebbing. This has been the main morphological feature identifying an HGPS cell line and has been used to determine the effectiveness of treatments for HGPS. The traditional method of measuring blebbing is a manual, blind count of the percentage of blebbed nuclei. However, this method has no standard criteria and is extremely time consuming. Sorting the nuclei into two categories, normal and blebbed, also obscures the fact that blebbing is not an either/or phenomenon, but varies continuously. The subjectivity and variability of the threshold for blebbed nuclei makes it impossible to compare values obtained by different counters. The need for an unbiased, quantitative method of measuring the degree of blebbing in a cell sample is clear.

In an effort towards solving this issue, we present an automated image analysis method using curvature as the primary measure of blebbing. We used a custom-written program to extract the boundaries of immuno-stained nuclei and calculate a curvature contour for each nucleus among other measures of shape. We found that several measures of the shape differentiate between HGPS and normal control cell lines. We focused on the most intuitive measure, the mean negative curvature (MNC), which is the average of all the concave curvatures on the boundary of a nucleus. MNC provides a continuous measure of blebbing that can be used in quantitative and statistical methods. We analyzed different seeding densities (Figure S3) and exposure times (data not shown) to demonstrate that MNC is also a consistent measure that does not vary significantly between experiments. The cluster analysis also shows that intensity does not affect the measured MNC (Figure S2). Thus MNC values can be compared between samples and experiments, unlike values obtained from the traditional blebbing count method. One caveat is that MNC is affected by pixel size and smoothing, thus care should be taken when comparing results from different laboratories. Of the other measures that strongly correlate with MNC, according to our clustering analysis (Figure S2), solidity should not be significantly affected by pixel size or smoothing and thus may be a viable alternative.

To better demonstrate the usefulness of this novel analysis, we treated HGPS and control cell lines with rapamycin, an mTOR pathway inhibitor that has been shown to improve nuclear morphology of HGPS cells, and with one of its analogues, RAD001, which is better tolerated by treated patients. The cells were treated for 7 weeks, stained with an anti-lamin A/C antibody, and analyzed using the program. Results of the treatment are presented through heat maps and box plots of MNC in Figure 3. Blinded blebbing counts were also performed (Figure 2), demonstrating that MNC agrees with the established method: RAD001 and rapamycin treated HGPS cells had significantly improved nuclear morphology to the same extent. Consistent with Cao et al. [15], we found that RAD001 promoted progerin degradation (Figure 2). In addition, we reported that RAD001 and rapamycin treatments decreased the DNA-damage induced 53BP1 foci formation in HGPS cells (Figure S4), likely through down-regulation of progerin. Consistent with our observation, previous studies have shown that rapamycin can inhibit the DNA-damage independent pseudo DNA-damage response, which might be caused by general over-activation in senescent cells [25, 26].

To demonstrate the sensitivity of this method, we used this program to distinguish between treatment doses that cannot be differentiated by the traditional bleb counting method (Figure 4). We treated HGPS and control cell lines with lower doses of RAD001 and used Student's t-test to show a statistically significant increase in MNC with lower doses. A blinded bleb count was unable to demonstrate any difference between the treatments (data not shown). In this treatment, we again noticed a dose-dependent change in nuclear area (Figures 3c and 4c). However, the same area change was observed in the treated normal control cell line, suggesting that this area change is primarily due to the action of mTOR inhibition and not an improvement of nuclear morphology in HGPS cells. We also showed the anti-hypertrophic effects of RAD001 in the early stages of treatment — within the first week at the indicated concentrations (Figure S5). This reduced cellular growth in the initial period of treatment and the area decrease of nuclei may be explained by the inhibition of the mTOR pathway. After the initial slowdown in growth during the first two weeks of treatment, rapamycin and RAD001 treated cells showed a greatly improved proliferation rate, better than their mock treated counterparts [15], which is consistent with the previously established role of rapamycin in preventing the loss of proliferative potential in cultured cells [27]. Notably, our multidimensional analysis of cell shapes provides unexpected hints into the mechanical aspects of mTOR inhibition: while RAD001 or rapamycin treatment decreases blebbing and nuclear size, they do not alter the eccentricity of the nuclear shape (Figure 3e). We expect that our high throughput, multi-dimensional measures will provide a solid foundation for developing mechanical models of the nucleus.

HGPS is a devastating and well-studied premature aging disease that currently has no effective treatment. HGPS also has strong connections with the general aging process. In both scenarios, the broad distribution of nuclear shape abnormality in a single population of cells hampers manual analysis. Our automated nuclear shape analysis software provides a high-throughput and easy-to-use method of quantifying nuclear morphology. Heat maps of curvature (Figures 1, 3, 4) allow us to directly visualize the broad distribution of nuclear blebbing in a large cell population. Comparing measures between samples allows us to assess treatment efficacy for HGPS and other age-related diseases. We use this method to demonstrate the potential of RAD001 as a treatment alternative for HGPS, being similarly effective to rapamycin. Our nuclear shape analysis provides an unbiased multidimensional fingerprint for a population of cells, which can be used to quantify treatment efficacy and analyze cellular aging.

## MATERIALS AND METHODS

### Cells, Cell Culture, and Treatments

Primary human dermal fibroblasts used in this study were obtained from the Progeria Research Foundation (PRF): two HGPS fibroblasts, HGADFN155 and HGADFN167, and a control normal fibroblast line, HGFDFN168. Fibroblasts were cultured in MEM medium (Invitrogen) supplemented with 15% FBS and 2 mM L-glutamine under 5% CO2 at 37°C. Normal and HGPS fibroblasts were replenished with fresh MEM medium containing 0.68 μM rapamycin/DMSO, or indicated concentration of RAD001/DMSO, every other day for up to 50 days. Control cells were treated with vehicle (DMSO) in MEM medium. Rapamycin (Sirolimus) was purchased from Sigma, and RAD001 (Everolimus) was obtained from Selleck.

### Immunofluorescence Staining

For immunofluorescence, cells were seeded in 4-well chamber slides. After fixation in 4% paraformaldehyde/PBS at room temperature for 15 min, cells were permeabilized with 0.5% Triton X-100/PBS at room temperature for 5 min, followed by an overnight incubation in the blocking solution (4% BSA/TBS) at 4°C. Cells were then stained with primary antibodies for 3 hours at room temperature on the following day. The primary antibodies used in this study were: a rabbit polyclonal antibody against progerin (custom peptide antibody, Yenzym); a goat anti-lamin A/C antibody (N-18, Santa Cruz); and a mouse anti-lamin A/C antibody (MAB3211, Chemicon). After primary antibody incubation, primary antibodies were detected with Alexa Fluor-labeled secondary antibodies (Invitrogen). Slides were mounted with Vectashield mounting medium containing DAPI and observed with a Zeiss fluorescence microscope. Images were taken using a 40x objective (about 100 nuclei per condition). Exposure times and acquisition settings were established at the beginning of each set of experiments and kept constant for all treatments.

### Extraction of Nuclei Boundaries

A custom-written MATLAB program was used to extract nuclei boundaries. In order to reduce image histogram variability both between and within images, we first used contrast-limited adaptive histogram equalization. An 8 × 8 grid of tiles, a clip limit of 0.02, and a Rayleigh distribution were employed. Next, we binarized the images using MATLAB's built-in thresholding function, which uses Otsu's method. Holes within bright regions were then filled, and regions that either overlapped the image boundary or were smaller than 800 square pixels were removed. In order to smooth and enlarge the regions, which correspond to nuclei, the images were morphologically eroded with a disk of radius 3 pixels and then morphologically dilated with a disk of radius 6 pixels. The convex hulls of these smoothed and enlarged regions were next calculated. These outer convex hulls were later used as an outer limit on the nuclei boundary positions. Next, the images were morphologically eroded with a disk of radius 2 pixels. The convex hulls of these smoothed and slightly enlarged regions were used to initialize an active contour based boundary extraction algorithm.

We next processed the original images for use by the active contour based boundary extraction algorithm. First, the brightness and contrast of the image was adjusted so that 1% of the pixels was saturated at the lowest intensity and 1% was saturated at the highest intensity. For each nucleus, any pixel outside of its outer convex hull, which was found as described above, was set to zero, and the brightness and contrast were again adjusted as before. We next calculated the binarization threshold using MATLAB's built-in thresholding function, but did not binarize the image. Instead, we nearly binarized the image by setting any pixel whose value was less than 70% of the threshold value to the lowest intensity and any pixel whose value was greater than 130% of the threshold value to the highest intensity. The remaining, non-saturated pixel intensities were then stretched to fill the entire intensity range. The holes in this gray-scale, nucleus image were next filled.

An active contour, or snake algorithm, was used to extract nuclei boundaries with sub-pixel resolution, as described in Xu and Prince [28]. The gradient vector flow (GVF) field of the processed, nucleus image was calculated. The inner convex hull of the nucleus, found as described above, was next interpolated and used as the initial position of the active contour. The contour, which is a polygon, was then repeatedly deformed 75 times and interpolated until the change in area from one set of deformations to the next was no more than 10 square pixels. Contours were not allowed to deform more than 50,025 times. The GVF, snake interpolation and snake deformation functions are from Xu and Prince [28]. (The following parameters were supplied to these three functions: number of GVF iterations, 80; d_min_, 0.5; d_max_, 2; tension or alpha, 0.02; rigidity or beta, 0.05; step size or gamma, 1; external force or kappa, 0.6.) The contour was interpolated a final time, resulting in an outputted polygon with sides of constant length (d_min_).

Some contours do not correspond to single nuclei, but rather are multiple, overlapped nuclei or are auto-fluorescent regions of cells. The user is next given an opportunity to remove such unwanted contours. Each analyzed image is sequentially displayed and polygons clicked on by the user are removed from further analysis.

### Analysis of Boundary Shape

We calculated the boundary curvature at each boundary point by fitting a circle to that boundary point and the two points 25 boundary points away from it. The curvature was then calculated as the reciprocal of the radius of that circle. Convex curvatures were kept positive, while concave curvatures were made negative. For each nucleus, the boundary point farthest from the centroid was labeled boundary point 0. When visualized with color, curvature values were cut-off such that magnitudes above a cut-off value were set to that cut-off value. For each nucleus, the number of invaginations was calculated by simply counting the number of boundary regions, of any length, where negative curvature was uninterrupted by positive curvature, and eccentricity was defined as the eccentricity of an ellipse with the same second-moments as the nuclear shape. The eccentricity of an ellipse describes how elongated the ellipse is; a circle would have an eccentricity of 0, and a line segment has an eccentricity of 1. We have previously similarly analyzed the shape of migrating amoebae [29]

### WST-1 Cell Proliferation Assay

A WST-1 cell proliferation assay (Roche) was used to analyze the effects of RAD001 on cellular growth. HGPS cell line HGADFN167 p12 and control cell line HGADFN168 p14 were seeded in separate standard 24-well plates at 10,000 cells in 500 μl fibroblast medium per well. Wells were treated with 0, 20, 60, 100 and 500 nM RAD001/DMSO in triplicate and the solvent controlled at 0.1% for all wells. The cells were then incubated with treatment for 72 hours. The medium was removed from each well and 500 μl of 10% WST-1 reagent in fibroblast medium was applied to each well following the incubation. Three blanks, consisting of 500 μl of 10% WST-1 reagent in fibroblast medium, were also created. The absorbance (450nm) of each well was read after 3 more hours of incubation using a SpectraMax M5^e^ plate reader (Molecular Devices), and the average absorbance of the blanks was subtracted from each measurement.

Cell numbers were calculated from the absorbance values using a standard curve established by repeating the experiment without treatment and seeding at 1000, 2000, 4000, 8000 and 16000 cells per well in duplicate. The percent survival was calculated for each sample by the equation: percent survival = 100 * (cell #)/(average cell # of 0 nM treatment), then averaged by treatment. The error was calculated using the standard deviation of the percent survivals of the 3 samples for each treatment.

### Statistics

Statistical analysis of MNC and other values was performed using the ttest2 function in MATLAB v7.12.0 that performs a Student's t-test. The parameters were set to a 1-tailed t-test, assuming unequal variances, and a p-value of 0.05 or less was considered significant.

## SUPPLEMENTARY FIGURES

Figure S1 Mean negative curvature (MNC) correlates with bleb counting. Images of 847 nuclei from HGPS and normal control cell lines were separately displayed to 3 members of the Cao lab. Each member selected the nuclei he or she considered to be blebbed using the standard criteria established in the Materials and Methods. The selections were saved and analyzed through a custom-written MATLAB program. The percentage of all nuclei with an MNC above a certain threshold that were labeled blebbed by the majority of counters (2 or more) is displayed for various MNC thresholds.

Figure S2 Covariance matrix (right) and hierarchical clustering plot (left) of 15 measures of nuclear shape and lamin A/C fluorescence intensity. Each box in the covariance matrix indicates the amount of correlation between two measures. High covariance is indicated in red (see color bar).

Figure S3 The plating density of the cells does not affect the curvature analysis. (**a**) HGPS fibroblast cells (HGADFN155-p15) were seeded at various cell densities (3000, 9000, and 27000 per well) in 4-well chamber slides and immuno-stained with anti-lamin A/C (N18, Santa Cruz) and DAPI. Images of the nuclear staining with DAPI and brightfield images are presented to help visualize the various cell densities. (**b**) Heat maps of curvature contours of 100 randomly selected nuclei for each density and sorted by MNC show a similar degree of blebbing at all cell densities. (**c**) Box plots of MNC created using MATLAB's boxplot function of nuclei imaged in the heat maps.

Figure S4 Genome stability is improved in rapamycin or RAD001 treated cells. (**a**) Fibroblast cells were immuno-stained with DAPI (blue) and TP53BP1 antibody (green); scale bar: 20 μm. (**b**) Percentage of TP53BP1 foci-positive cells in Rad100, Rad500, rapamycin and mock treated HGPS cells. Mock: mock treatment; Rap: 0.68 μM rapamycin treatment; Rad100: 0.1 μM RAD001 treatment; Rad500: 0.5 μM RAD001 treatment. The percentage of cells with no TP53BP1 staining is shown in blue, the percentage of cells with one TP53BP1 is in red, and percentage of cells with more than one TP53BP1 foci is shown in yellow.

Figure S5 A cell proliferation assay shows that all treatments of RAD001/DMSO at indicated dosages for both normal (blue) and HGPS (red) fibroblast cell lines had similarly reduced growth compared to the mock treatments. All treatments were controlled for DMSO at 0.1% and percent survival calculated relative to the cell numbers of the mock treatments.
