# Supplementary figures and images for: Automated image analysis of nuclear shape: What can we learn from a prematurely aged cell?
Source: Aging (Albany NY). 2012 Feb 16;4(2):119–32. doi: 10.18632/aging.100434 (PMC3314174; doi:10.18632/aging.100434)

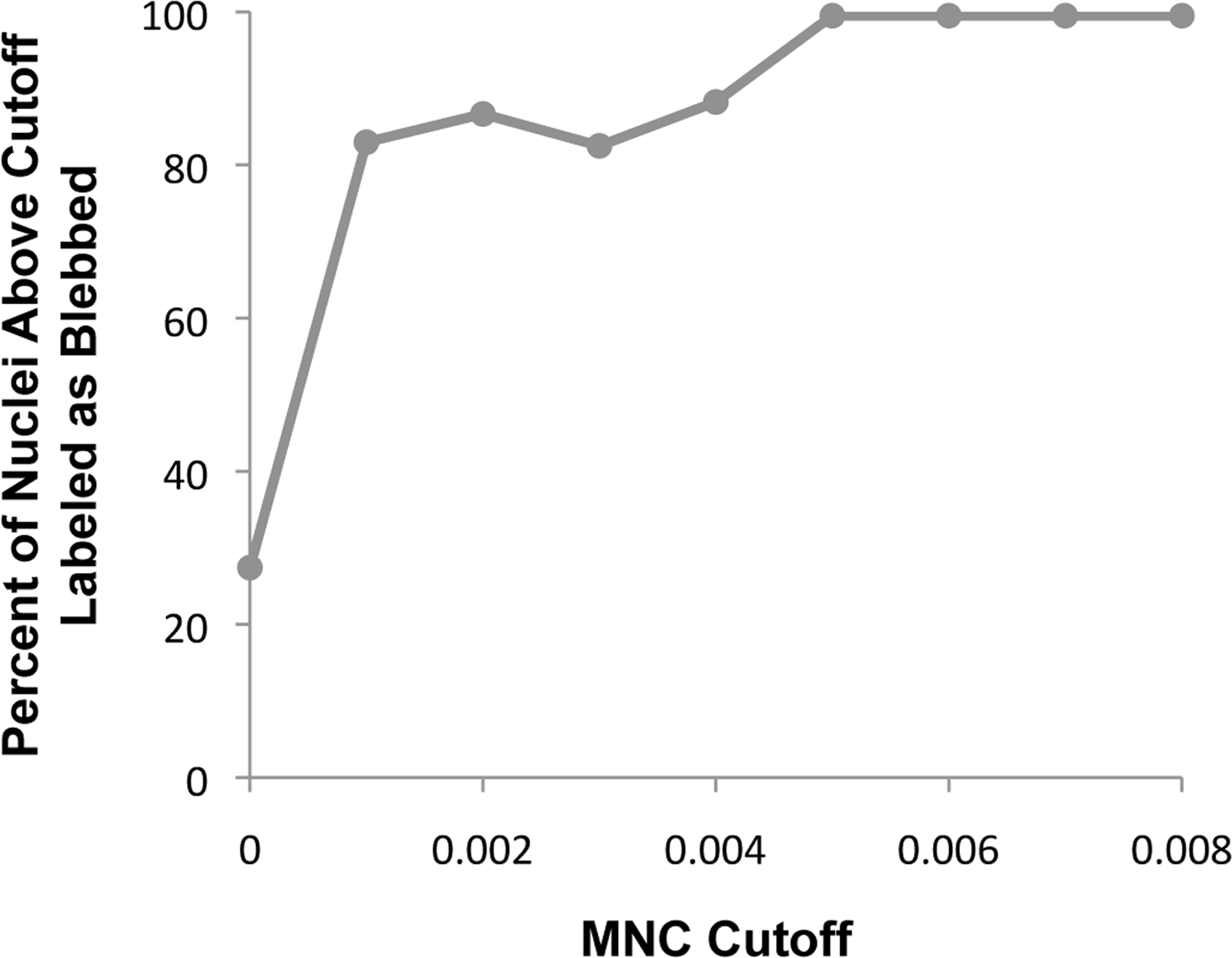

Supplement: Figure S1 — Mean negative curvature (MNC) correlates with bleb counting. Images of 847 nuclei from HGPS and normal control cell lines were separately displayed to 3 members of the Cao lab. Each member selected the nuclei he or she considered to be blebbed using the standard criteria established in the Materials and Methods. The selections were saved and analyzed through a custom-written MATLAB program. The percentage of all nuclei with an MNC above a certain threshold that were labeled blebbed by the majority of counters (2 or more) is displayed for various MNC thresholds. [file aging-04-119-s001.tif]

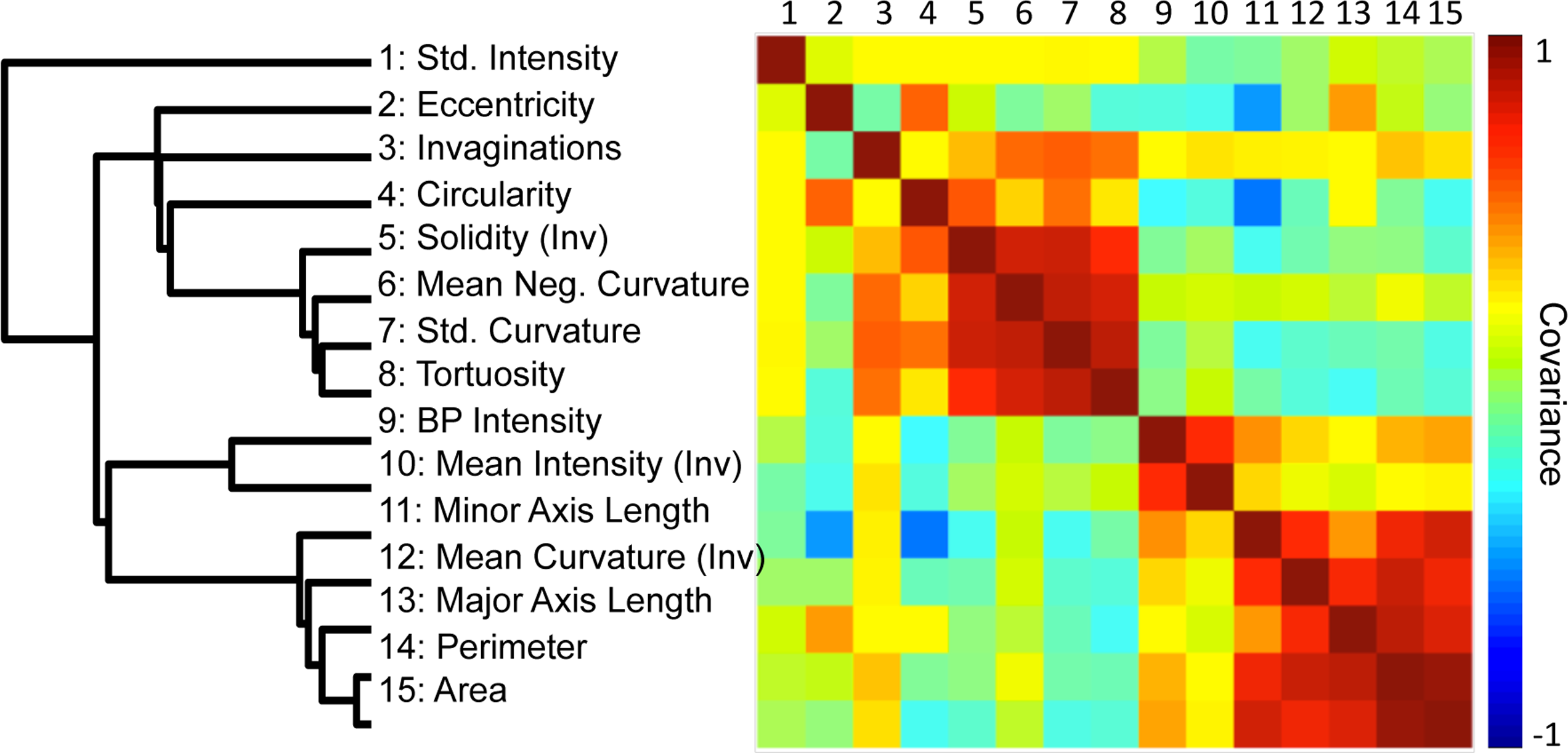

Supplement: Figure S2 — Covariance matrix (right) and hierarchical clustering plot (left) of 15 measures of nuclear shape and lamin A/C fluorescence intensity. Each box in the covariance matrix indicates the amount of correlation between two measures. High covariance is indicated in red (see color bar). [file aging-04-119-s002.tif]

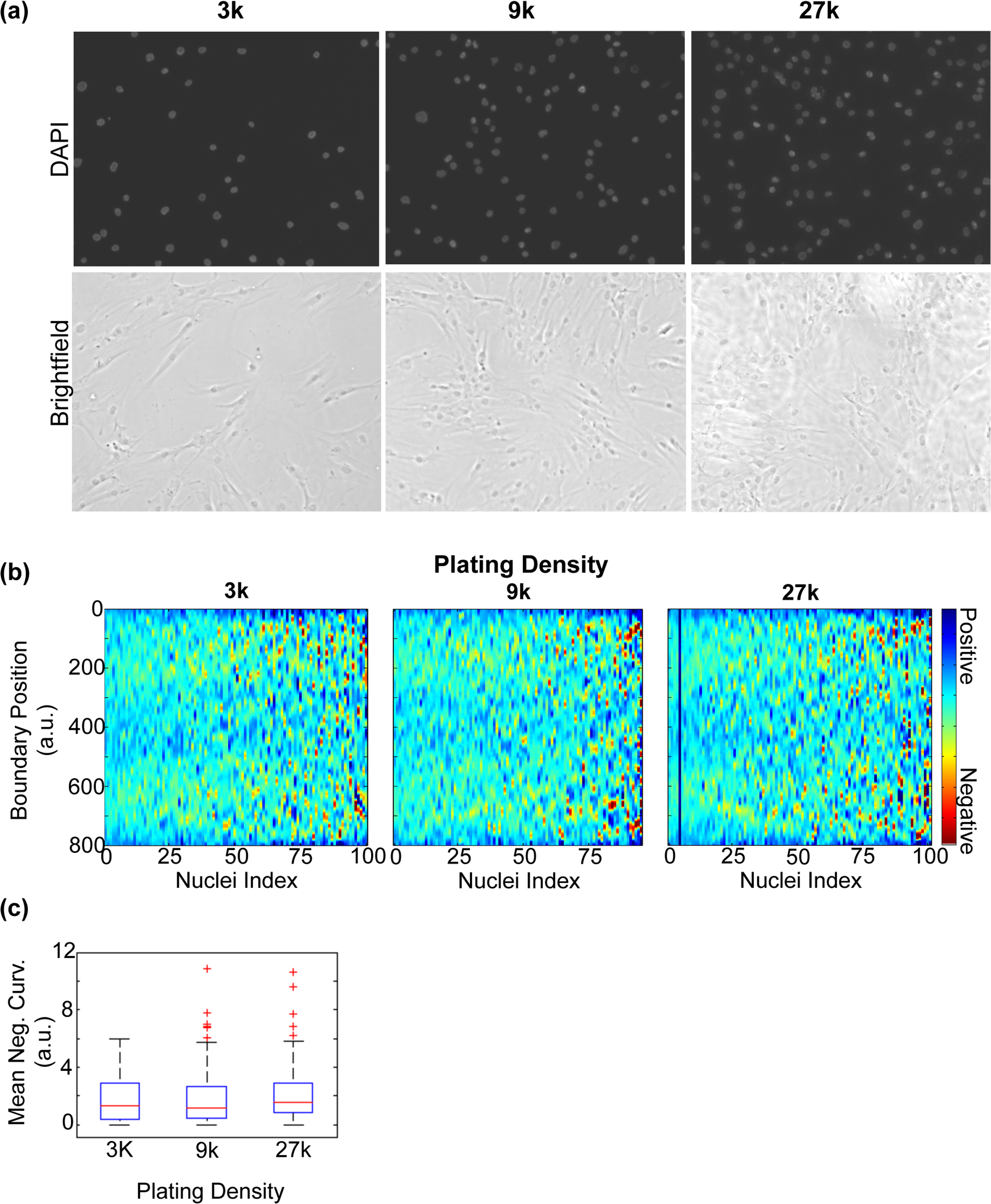

Supplement: Figure S3 — The plating density of the cells does not affect the curvature analysis. (a) HGPS fibroblast cells (HGADFN155-p15) were seeded at various cell densities (3000, 9000, and 27000 per well) in 4-well chamber slides and immuno-stained with anti-lamin A/C (N18, Santa Cruz) and DAPI. Images of the nuclear staining with DAPI and brightfield images are presented to help visualize the various cell densities. (b) Heat maps of curvature contours of 100 randomly selected nuclei for each density and sorted by MNC show a similar degree of blebbing at all cell densities. (c) Box plots of MNC created using MATLAB's boxplot function of nuclei imaged in the heat maps. [file aging-04-119-s003.tif]

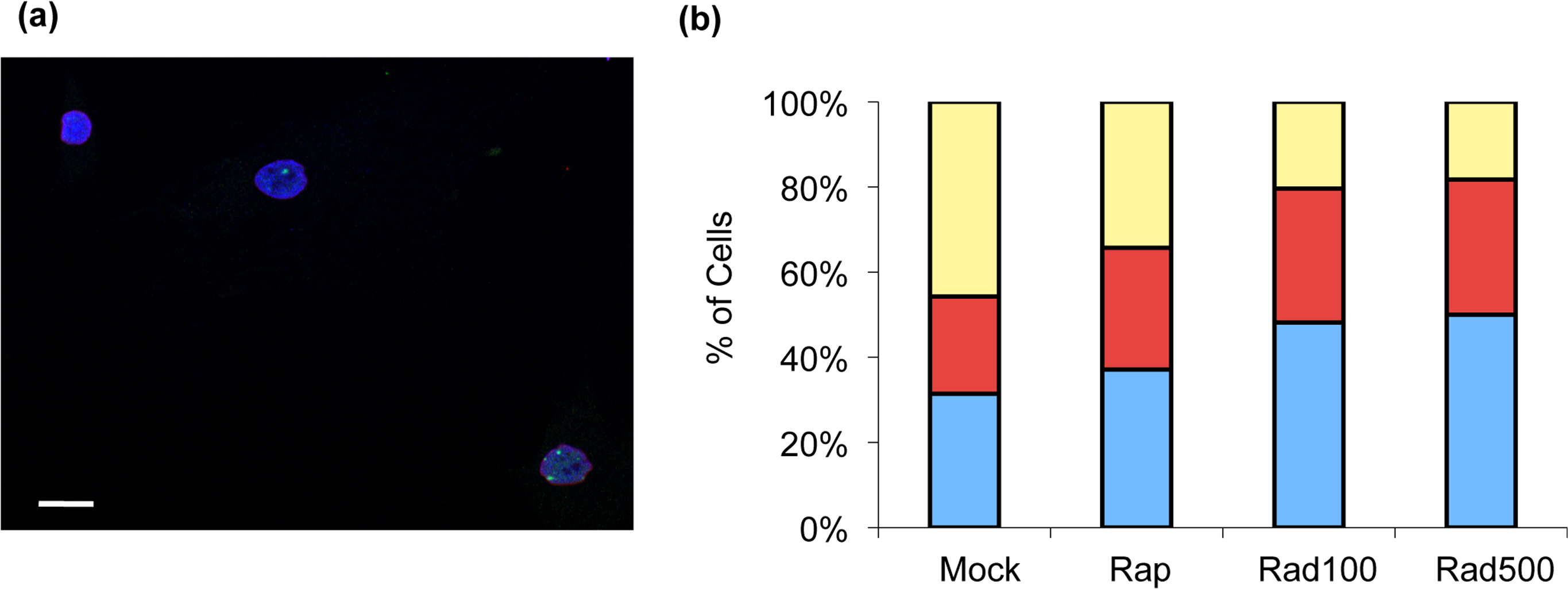

Supplement: Figure S4 — Genome stability is improved in rapamycin or RAD001 treated cells. (a) Fibroblast cells were immuno-stained with DAPI (blue) and TP53BP1 antibody (green); scale bar: 20 μm. (b) Percentage of TP53BP1 foci-positive cells in Rad100, Rad500, rapamycin and mock treated HGPS cells. Mock: mock treatment; Rap: 0.68 μM rapamycin treatment; Rad100: 0.1 μM RAD001 treatment; Rad500: 0.5 μM RAD001 treatment. The percentage of cells with no TP53BP1 staining is shown in blue, the percentage of cells with one TP53BP1 is in red, and percentage of cells with more than one TP53BP1 foci is shown in yellow. [file aging-04-119-s004.tif]

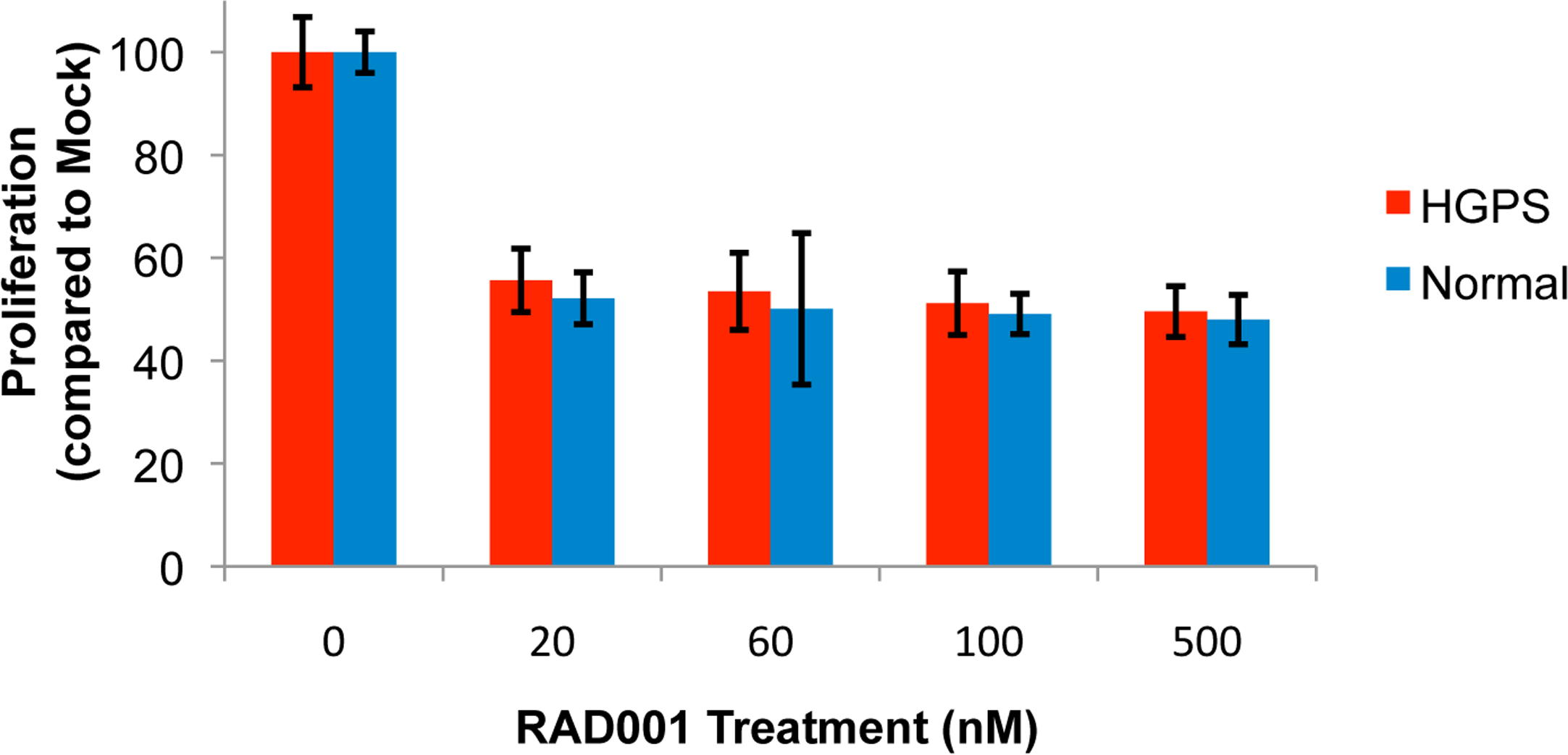

Supplement: Figure S5 — A cell proliferation assay shows that all treatments of RAD001/DMSO at indicated dosages for both normal (blue) and HGPS (red) fibroblast cell lines had similarly reduced growth compared to the mock treatments. All treatments were controlled for DMSO at 0.1% and percent survival calculated relative to the cell numbers of the mock treatments. [file aging-04-119-s005.tif]
